# Intrahepatic umbilical vein: An underutilized access site for intrauterine transfusion

**DOI:** 10.1002/pmf2.70176

**Published:** 2025-11-14

**Authors:** Kenneth J. Moise, Erin E. Moise

**Affiliations:** ^1^ Department of Women's Health Dell Medical School at The University of Texas Austin Texas USA; ^2^ Comprehensive Fetal Care Center at Dell Children's Medical Center Austin Texas USA

**Keywords:** cordocentesis, fetal anemia, intrahepatic umbilical vein, intraperitoneal transfusion, intrauterine transfusion, intravascular transfusion

## 1 BACKGROUND

Intrauterine transfusion (IUT) represents the earliest and most successful form of fetal intervention. Accepted indications include the correction of fetal anemia in cases of hemolytic disease of the fetus and newborn, fetal parvovirus infection, twin anemia‐polycythemia sequence, and fetomaternal hemorrhage. The initial intraperitoneal approach to IUT was later replaced by the intravascular IUT when it was demonstrated that the fetal circulation could be safely accessed.

**FIGURE 1:**
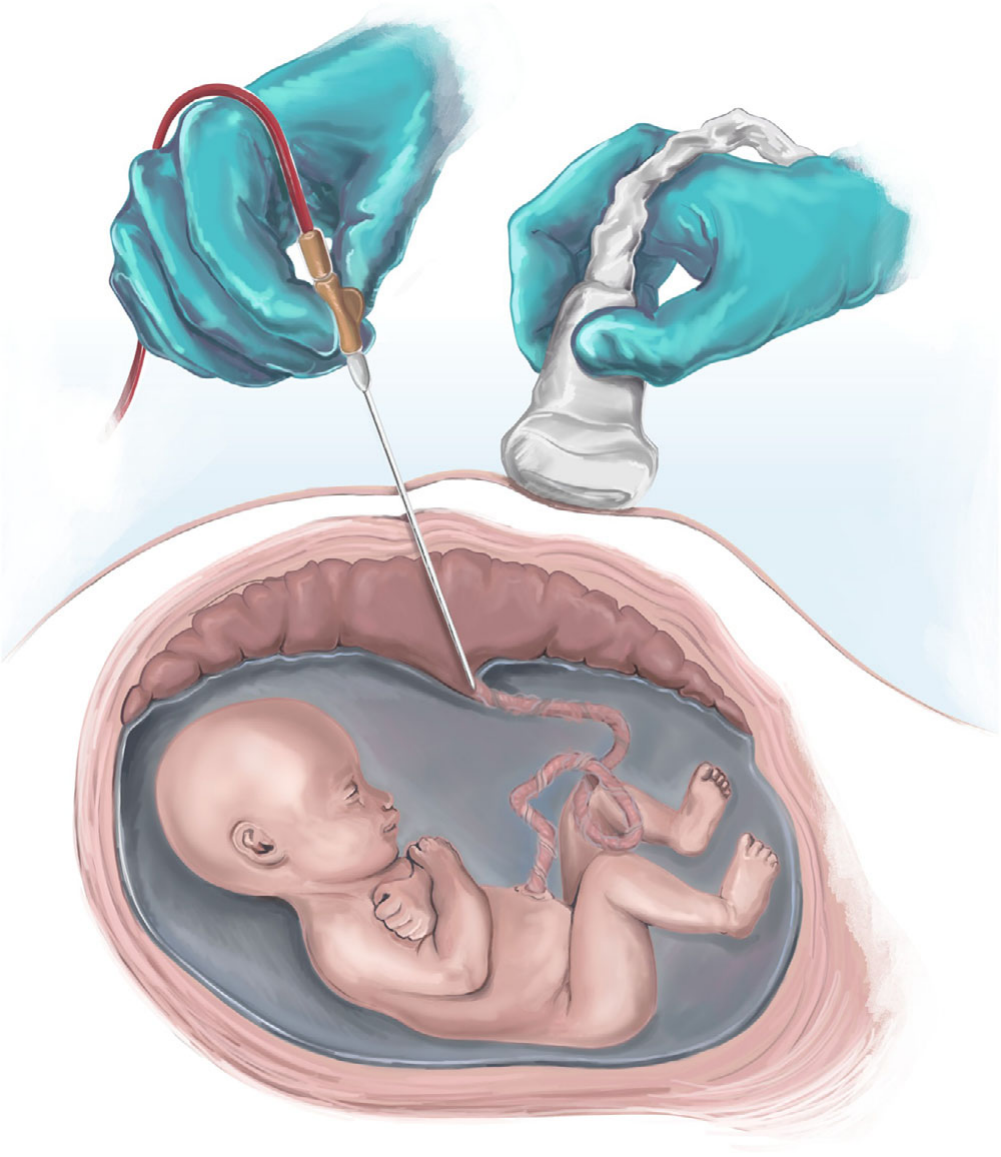
Transplacental approach to cord insertion with an anterior placentation.

**FIGURE 2:**
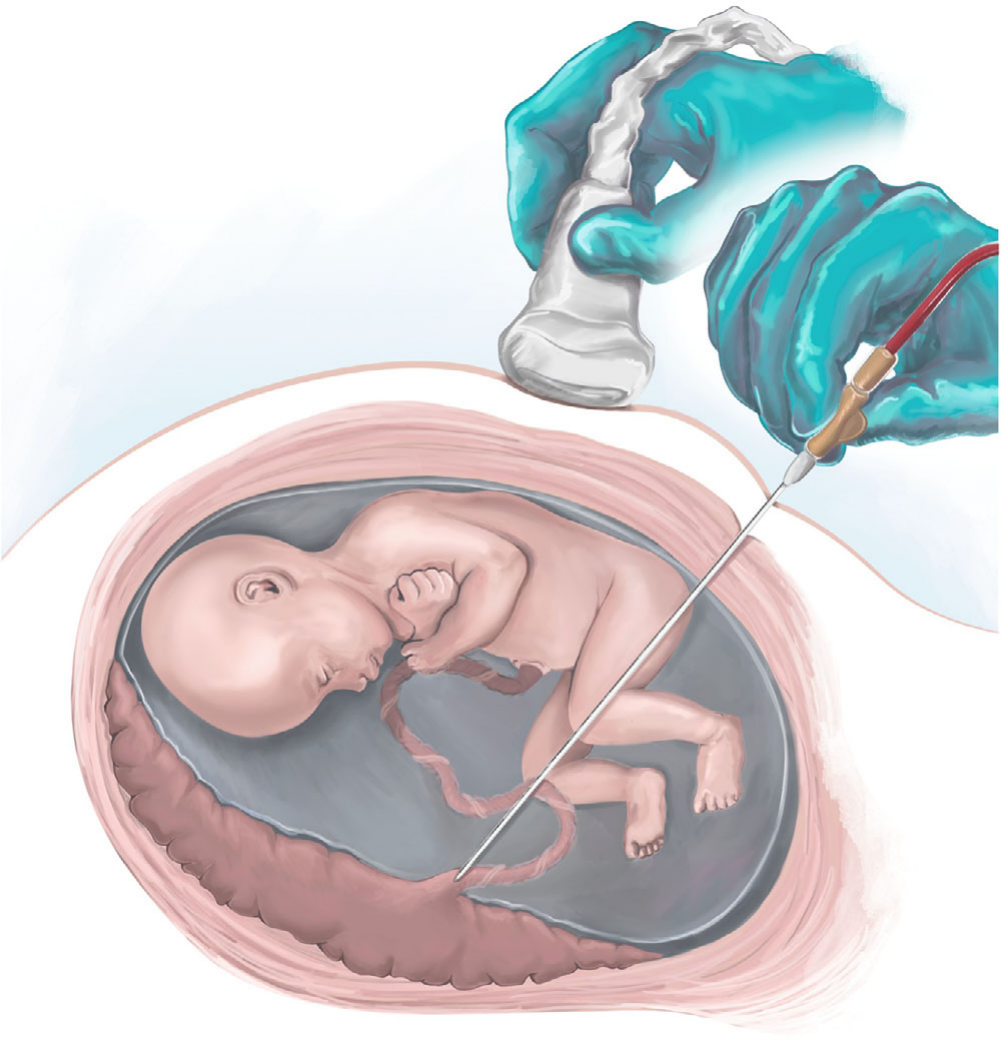
Transamniotic approach to cord insertion with posterior placentation.

**FIGURE 3:**
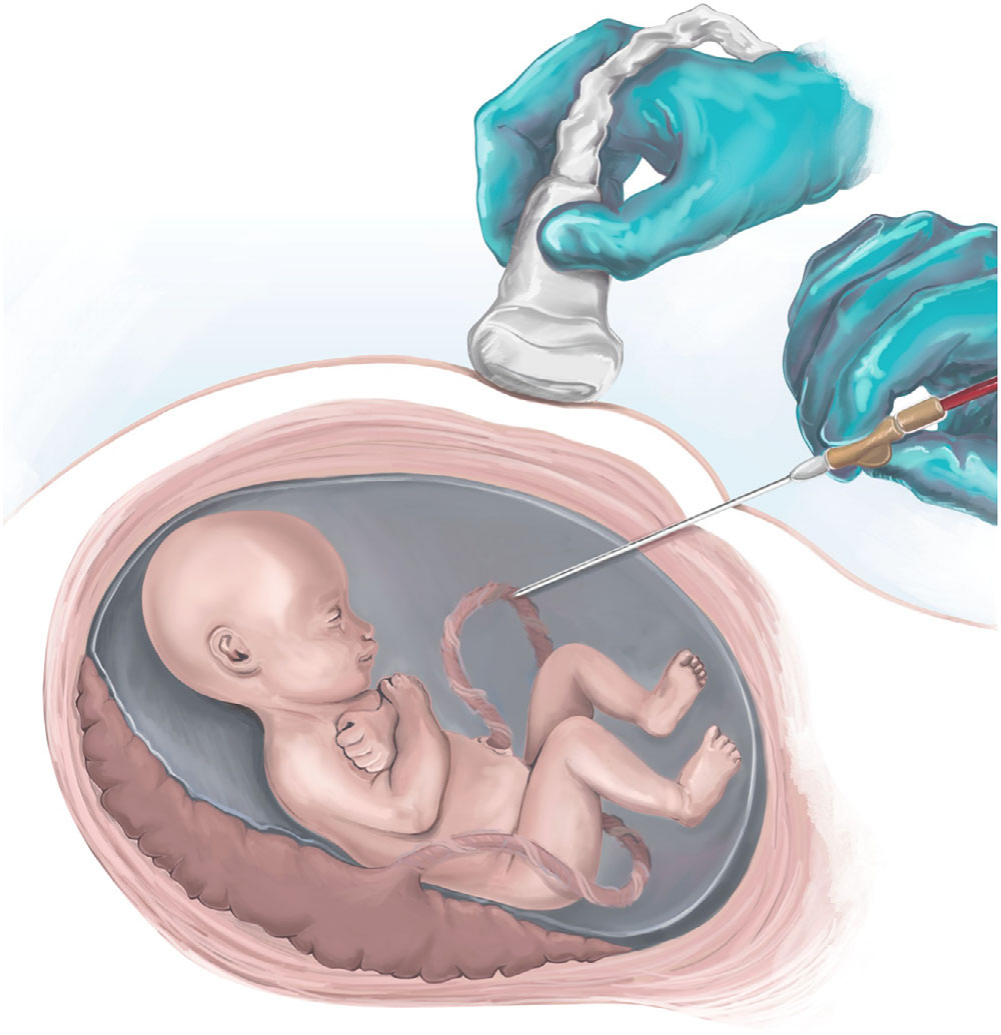
Transamniotic approach to floating loop of cord with posterior placentation.

**FIGURE 4:**
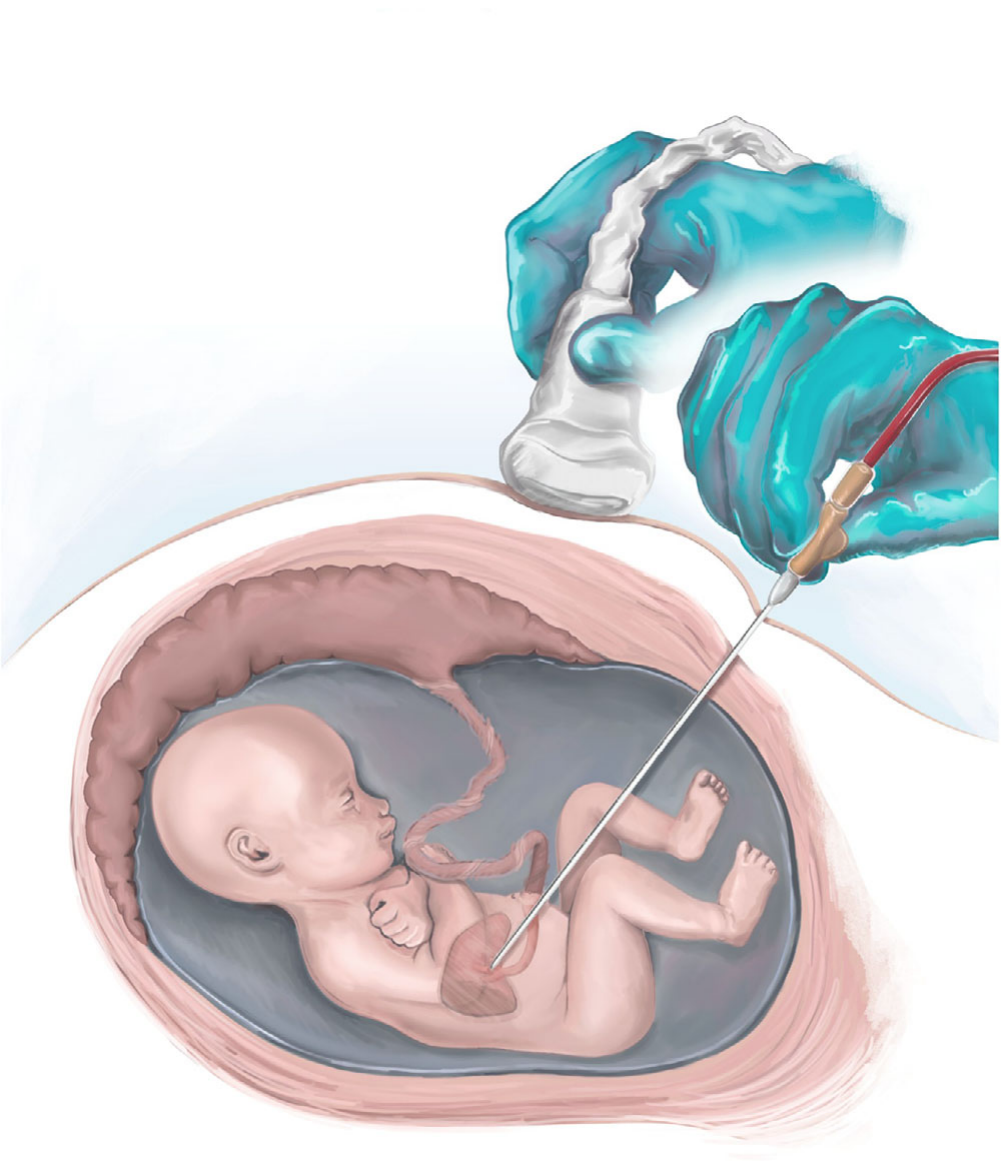
Transamniotic approach to intrahepatic portion of the fetal umbilical vein with anterior placentation.

Intravascular access to the fetal circulation can be undertaken through ultrasound‐guided umbilical venous puncture at the site of the cord insertion into the placenta, a free‐floating loop of cord or the intrahepatic portion of the umbilical vein (IHUV) (Figures 1, 2, 3, 4).

Bang et al. [1] were the first to describe an intravascular IUT using the IHUV. Subsequently, Nicolini et al. [2] documented the safety of using the IHUV for fetal vascular access in a series of 142 sampling procedures and 72 IUTs. Previously, anterior placentation required a direct transplacental approach to obtain UV access; now most fetal centers across Europe have adopted the use of the IHUV in cases where the placenta was localized to other areas of the uterus. A recent Delphi survey from 24 different countries (41% from North America) found that one‐third of centers use the umbilical vein at its insertion into either an anterior or a posterior placenta as the primary target for access followed by the umbilical vein in a floating loop of cord [3].

## 2 ADVANTAGES

Anatomically, the umbilical vein tracks through the fetal liver before joining with the left and right hepatic portal veins and continuing as the ductus venosus. The fetal umbilical arteries originate from the internal iliac vessels before coursing superiorly around the fetal bladder and then entering the site of the umbilical cord insertion into the fetal abdominal wall. Thus, the umbilical arteries are not located proximate to the IHUV. Umbilical artery puncture by cordocentesis has been associated with a higher risk of fetal bradycardia. This is thought to be related to the increased muscularis layer of the vessel wall as compared to the umbilical vein with a resulting tendency for vasospasm and vagal activation. In a study of 1678 IUTs, Zwiers et al. [4] defined severe procedure‐related complications as preterm delivery or premature rupture of membranes within 7 days of IUT, intrauterine infection, fetal or neonatal death, or fetal distress requiring delivery by emergency Cesarean section within 24 h after IUT. Umbilical arterial puncture at IUT was associated with a 10.8‐fold (95% confidence interval, 3.5–33.6X) increased incidence of non‐lethal perioperative complications. Using the IHUV as the site of vascular access also has the potential advantage of stabilization of the procedure needle since the surrounding hepatic parenchyma offers resistance to needle movement. In the Dionysus observational cohort study, perioperative complications occurred in 2% of cases using the IHUV as compared to a frequency of 6.9% for placental cord insertion and 13.3% when a free loop of umbilical cord was used [5]. When the IHUV is used for IUT, any leakage of blood that occurs can be readily absorbed from the peritoneal cavity. Intraperitoneal bleeding was noted in 2.3% of 72 cases in one series; all ultrasound evidence of blood extravasation had disappeared within 48 h [2]. Once the intravascular transfusion in the IHUV has been completed, some experts will redirect the operative needle into the peritoneal cavity and infuse additional donor red cells in an effort to prolong fetal hemoglobin stabilization between procedures [6]. Finally, use of the IHUV has the potential to avoid an increase in maternal titer due to an anamnestic response, especially in the case of anterior placentation requiring a transplacental approach. Using maternal serum alpha‐fetoprotein as a marker for fetomaternal hemorrhage, Nicolini et al. [7] found an increase of 50% in 65% of cases of IUT with an approach through an anterior placenta as compared to a rate of 17% with posterior placentation. When the volume of fetomaternal hemorrhage was calculated to be in excess of 1 mL, five of seven patients had a subsequent increase in titer of >150%. The authors also detected an increased rate of decline in the fetal hemoglobin/day between transfusions in these patients.

## 3 SAFETY

The IHUV approach to IUT requires that the track of the needle pass through liver parenchyma. Nicolini et al. [2] measured hepatic enzymes in 13 fetuses undergoing IUT using the IHUV and compared the changes to 13 fetuses undergoing IUT using the cord insertion. Only gamma glutamyl transpeptidase was observed to increase in the IHUV group (−8% to +21%) as compared to the cord insertion group (−11% to +6%; *p* = 0.01). At the time of the subsequent IUT, the small increase was less than that observed in the cord insertion group. Concerns may arise regarding an increased fetal stress response to the noxious stimulus of the IHUV approach. A comparison of cortisol, beta endorphin, and noradrenaline levels in fetuses undergoing IUT using the IHUV versus the placenta cord insertion failed to reveal any significant differences between the two approaches [8].

## 4 METHODOLOGY

The following is the approach we have used at our center in the use of the IHUV for intrauterine transfusions.

**FIGURE 5:**
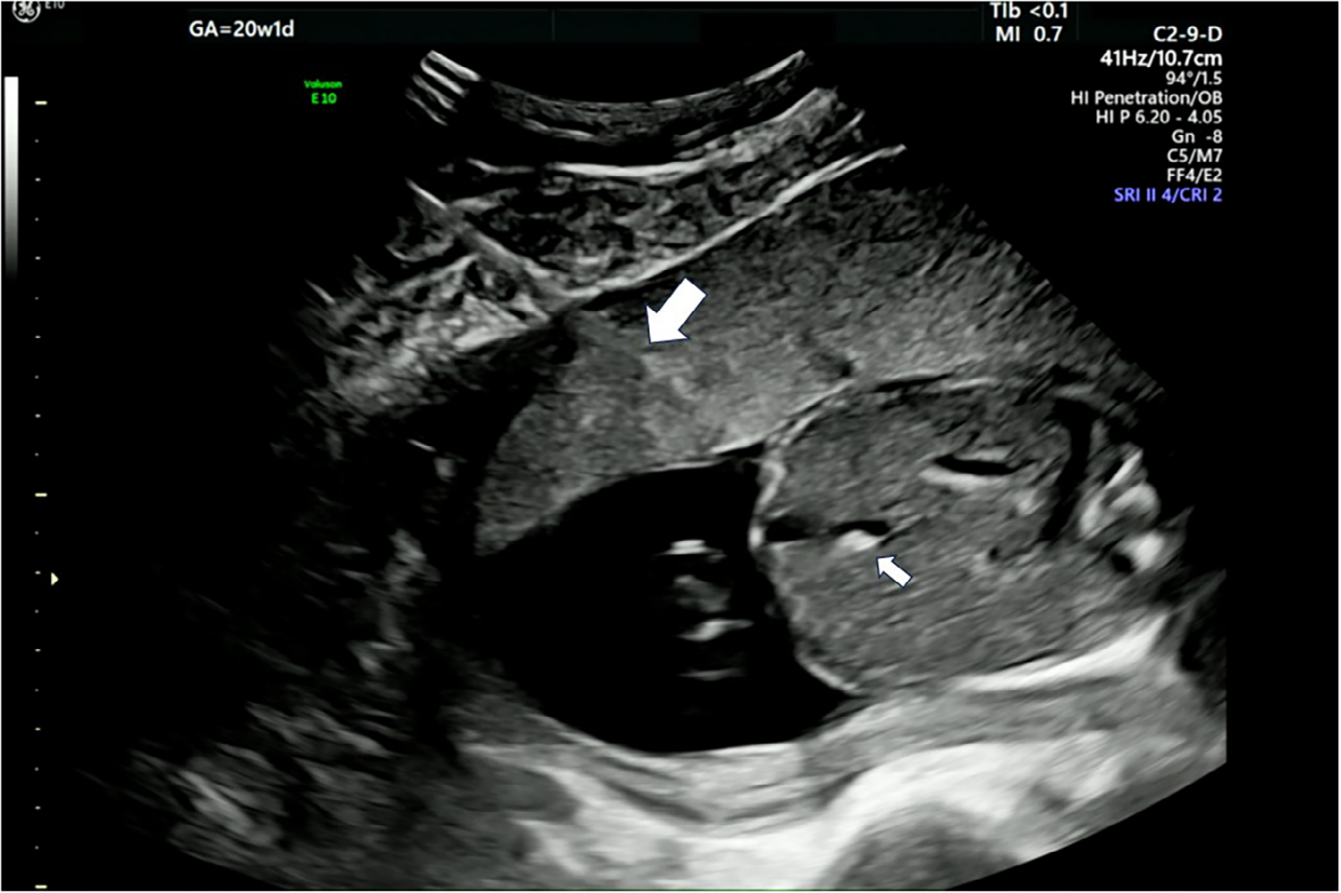
Ultrasound image of transplacental approach to the fetal IHUV for IUT at 20 1/7 weeks’ gestation for fetal parvovirus infection (MCA‐PSV: 2.16 MoM; initial fetal hematocrit: 10%). Large arrow indicates procedure needle path; small arrow indicates tip of needle in the IHUV. IHUV, intrahepatic umbilical vein; IUT, intrauterine transfusion; MCA‐PSV, middle cerebral artery peak systolic velocity; MoM, multiples of the median.

IUTs using the IHUV are similar to other intravascular IUTs with some nuances. We do not routinely use a pre‐procedure paralytic agent administered by fetal intramuscular injection, although such an approach might be beneficial when an operator is first attempting the use of the IHUV. Ultrasound visualization of the IHUV with color Doppler will aid in planning the approach. The fetal gallbladder and stomach should be identified and avoided. A 22‐gauge needle is used for gestational ages of less than 22 weeks while a 20‐gauge needle is used for more advanced gestational ages. The needle should be advanced under ultrasound guidance into the intra‐amniotic space while briefly pausing just adjacent to the fetal abdominal wall to realign the needle with the target IHUV (see video of procedure). It should then be advanced rapidly as a slow advance of the needle may cause the fetus to move and compromise vascular access. If access is not obtained, the needle should be withdrawn to just inside the fetal abdominal wall and a new trajectory chosen since the liver parenchyma will prevent lateral movement of the needle. We administer an intravascular fetal paralytic agent after the initial blood sample is obtained for fetal hematology. The fetal heart rate can be monitored during the procedure by visualization of the fetal abdominal aorta. Access to the IHUV is easier when the fetus is spine down; however, even when the fetus is spine up or spine transverse, a more lateral approach can be used for access even though this requires traversing a greater amount of hepatic tissue (Figure 5).

## 5 CONCLUSION

The IHUV approach to IUT is favored by many fetal interventionists as a first‐line approach in cases where the placenta is not located on the anterior side of the uterus. In those centers that use access at the cord insertion into a posterior placenta, IHUV remains a viable alternative in cases when the fetus obstructs the original vascular access site. Other indications and the advantage of the use of the IHUV in these cases are described in Table 1. Use of a floating loop of cord for IUT access should be limited based on a higher risk for procedure‐related complications.

**TABLE 1:** Possible indications for the use of IHUV for IUT.

Indication	Advantage
Early gestational age	IHUV often dilated in cases of fetal anemia IHUV easier to visualize as a target if fetus overlies posterior cord insertion
Fetal hydrops	Easier visualization than cord insertion especially with anterior hydropic placenta
Oligohydramnios	Easier visualization than cord insertion
Multiple gestation	Ease of identification of which fetus being transfused Less manipulation of needle through membranes as compared to targeting a floating cord
Unable to visualize cord insertion	Preferred over floating loop access since this is associated with a higher risk of complications
Fetal thrombocytopenia	Potential for less post‐operative bleeding since surrounding hepatic tissue may tamponade the puncture site
Desire to perform combined IVT/IPT procedure	Single amniotic fluid and fetal puncture
High maternal antibody titer	Less often associated with rise in titer if placenta can be avoided Less decline in fetal hemoglobin between early IUTs Increased chance for lower titer in a subsequent pregnancy

Abbreviations: IHUV, intrahepatic umbilical vein; IPT, intraperitoneal transfusion; IUTs, intrauterine transfusions; IVT, intravascular transfusion.

## CONFLICT OF INTEREST STATEMENT

The authors declare no conflicts of interest.

## FUNDING INFORMATION

The authors received no specific funding for this work.

## ETHICS STATEMENT

As a commentary, the authors did not submit an application for ethical approval to the Dell Medical School–University of Texas Institutional Review Board.

A patient consent was obtained and uploaded to the journal website for the anonymized use of a video image as Figure #5 and the embedded video file to demonstrate the procedure.

## Supporting information



Transplacental access for the intrahepatic umbilical vein at 20 5/7 weeks’ gestation.
